# Supplementary material for: Polyketide synthase gene clusters in the ericoid mycorrhizal fungus Oidiodendron maius: genome-wide prediction, expression profiling, and functional characterization of knockout mutants
Source: Front Plant Sci. 2026 Sep 16;17:1924430. doi: 10.3389/fpls.2026.1924430 (PMC13623903; doi:10.3389/fpls.2026.1924430)
Supplement: Supplementary file 1 [file SupplementaryFile1.zip › Supplementary Materials/Supplementary Folder 1.zip!/Oidma1_AssemblyScaffolds_Repeatmasked/knownclusterblast/cluster26/jgi.p_Oidma1_166268_mibig_hits.html]

| MIBiG Protein | Description | MIBiG Cluster | MiBiG Product | % ID | % Coverage | BLAST Score | E-value |
| --- | --- | --- | --- | --- | --- | --- | --- |
| CAE17522.1 | NDP-3-ketoreductase | BGC0000210 | Chromomycin\_A3\_biosynthetic\_gene\_cluster | 31 | 52.6054590571 | 79 | 1e-14 |
| WP\_052390555.1 | oxidoreductase | BGC0001308 | Simocyclinone\_D9\_/\_simocyclinone\_D10\_/\_simocyclinone\_D11 | 33 | 34.9875930521 | 73 | 7.2e-13 |
| ACB46498.1 | sugar\_3-ketoreductase | BGC0000082 | Kijanimicin\_biosynthetic\_gene\_cluster | 31 | 33.2506203474 | 70 | 4.6e-12 |
| CAA09647.1 |  | BGC0000227 | Granaticin\_biosynthetic\_gene\_cluster | 31 | 40.6947890819 | 69 | 7.9e-12 |
| AGI99504.1 | sugar\_3-ketoreductase | BGC0001004 | Lobophorin\_biosynthetic\_gene\_cluster | 30 | 40.6947890819 | 64 | 4.3e-10 |
| AGC09477.1 | LobD7 | BGC0001183 | Lobophorin\_biosynthetic\_gene\_cluster | 30 | 40.6947890819 | 64 | 4.3e-10 |
| BAD20760.1 | dehydrogenase | BGC0000702 | Kanamycin\_biosynthetic\_gene\_cluster | 29 | 50.3722084367 | 63 | 5.7e-10 |
| CAE46940.1 | putative\_dehydrogenase\_kanC | BGC0000704 | Kanamycin\_biosynthetic\_gene\_cluster | 33 | 34.9875930521 | 63 | 5.7e-10 |
| ABX00611.1 | LmbZ | BGC0000907 | Lincomycin\_biosynthetic\_gene\_cluster | 27 | 48.8833746898 | 63 | 7.4e-10 |
| AFJ52691.1 | sugar\_3-ketoreductase | BGC0001073 | Kosinostatin\_biosynthetic\_gene\_cluster | 31 | 50.3722084367 | 63 | 7.4e-10 |
| CAF31590.1 | oxidoreductase | BGC0000705 | Kanamycin\_biosynthetic\_gene\_cluster | 29 | 50.3722084367 | 62 | 9.7e-10 |
| ABX71127.1 | Lct44 | BGC0000238 | Lactonamycin\_biosynthetic\_gene\_cluster | 31 | 34.7394540943 | 62 | 1.6e-09 |
| ABV97147.1 | oxidoreductase\_domain\_protein | BGC0000137 | Rifamycin\_biosynthetic\_gene\_cluster | 31 | 29.5285359801 | 57 | 5.3e-08 |
